# Knowledge, attitudes and practice of infection prevention and control in the CT suite

**DOI:** 10.1186/s12913-023-09779-9

**Published:** 2023-07-08

**Authors:** Dania Abu Awwad, Suzanne Hill, Sarah Lewis, Yobelli Jimenez

**Affiliations:** Discipline of Medical Imaging Science, Faculty of Medicine and Health, The University of Sydney, Camperdown, Australia

**Keywords:** Infection control, Computed tomography, Radiographers, Nurses, Contrast injectors, Radiology department

## Abstract

**Background:**

Infection, prevention, and control (IPC) practices are essential to protect patients and staff within healthcare facilities. Radiology departments cater to both inpatients and outpatients, and breaches of IPC practice have led to outbreaks of disease within healthcare facilities. This study aims to examine the knowledge, attitudes and practice (KAP) of computed tomography (CT) radiographers and nurses in their infection, prevention, and control (IPC) practice. The KAP components focuses on the CT environment, contrast injector use, and workplace factors that impact IPC practice.

**Methods:**

A cross-sectional KAP survey was distributed online to Australian CT radiographers and radiology nurses across different institutions. The survey covered demographics, each KAP component, and workplace culture. Spearman’s correlation was used to compare KAP scores. Kruskal–Wallis test was used to compare the KAP scores between demographic categories, and Chi Square was used to compare demographic data with workplace culture.

**Results:**

There were 147 respondents, 127 of which were radiographers and 20 were nurses. There was a moderate positive correlation between knowledge and attitude for radiographers (rho = 0.394, *p* < 0.001). Radiographers also had a moderate positive relationship between attitudes and practice (rho = 0.466, *p* < 0.001). Both radiographers and nurses scored high in the knowledge section of the survey, but nurses had statistically significant higher practice scores than radiographers (*p* = 0.014). CT radiographers who had an IPC team in their workplace or worked in public hospitals, had statistically significant higher attitudes and practice scores. Age, education, and years of experience did not impact on KAP scores.

**Conclusion:**

The study found that radiographers and nurses had a good baseline knowledge of standard precautions. IPC teams and continued training is important to positively influence knowledge and attitudes of health professionals towards IPC practice. The KAP survey was a useful tool to assess the knowledge, attitudes, and practice on IPC of CT radiographers and nurses and identified areas for education, interventions, and leadership.

## Introduction

Healthcare associated infections (HAI) are infections that are the direct result of procedures or examinations within healthcare facilities [1]. HAI are preventable, and proper infection control practices by healthcare workers help to reduce the risk [1]. All healthcare workers, including radiographers and nurses, must apply standard precautions to all patients [1, 2]. Breaches of standard precautions and aseptic technique have led to outbreaks of disease within healthcare facilities [1].

Radiology departments cater to both inpatients and outpatients, and radiographers are in close contact of many patients as radiology is a high caseload healthcare area [3]. Part of a radiographer’s role is to ensure patient safety by adhering to appropriate infection control practices to reduce the risk of HAI [4]. The demand for computed tomography (CT) services has continued to increase, and often involves intravenously injecting iodine contrast during scans [5]. Catheters, needleless connectors and contrast injectors used in CT imaging pose an infection, prevention, and control (IPC) risk due to the invasiveness of the procedures, and connections to power injectors and tubing have been found to be a commonly contaminated area [6, 7]. Education and training are important to reduce catheter-related infections and avoid transmission of infections from healthcare workers [2].

Previous studies have explored IPC risks in radiology departments [2, 3, 8], however, knowledge, attitudes, and practice (KAP) of IPC among radiographers and radiology nurses have not been assessed together, and there are limited studies that have focused on the CT suite or injectors [9]. A study by Abdelrahman et al., [10] only assessed radiographers’ IPC knowledge, while Alnahhal et al., [11] assessed knowledge and practice without exploring the attitudes of radiographers. This is an important consideration, as a review of nursing infection control practices revealed that nurses had a good understanding of standard practice, but poor adherence because of their perception of risk [12]. In Australia, recent research has focused on the impact of COVID-19 on workload and wellbeing [13, 14] but there has been limited explorations of IPC practices specific to CT and contrast injectors. Automatic contrast injectors pose IPC risks as they are a frequently touched surface that deliver substances directly into the patient’s bloodstream via a patient’s catheter [3]. The increased risk of contamination that patients and staff face require exploration of current IPC practice in CT, as well as current awareness and compliance with national guidelines to ensure the safety of both patients and staff.

## Aims

This study aims to examine the KAP of CT radiographers and nurses in their IPC practice. Additionally, the study aims to focus on the CT environment, contrast injector use, and workplace factors that impact IPC practice.

## Methods

### Study design

A cross-sectional KAP survey was previously developed using National Health and Medical Research Council (NHMRC) guidelines and appropriated for the CT environment and equipment (Hill et al. 2023. Infection, prevention, and control in computer tomography: methodological principles for a national survey in knowledge, attitudes, and practices. [Manuscript submitted for publication]). This survey was used in the current study and distributed to Australian CT radiographers and radiology nurses from September to December 2022. The survey was shared using Research Electronic Data Capture (REDCap) [15] using email invitations and electronic newsletter advertisements with the Australian Society of Medical Imaging and Radiation Therapy (ASMIRT). A snowballing effect was used to further distribute the survey, where the survey invitation encouraged recipients to forward the invitation to other radiographers and radiology nurses.

### Ethical considerations

Ethical approval was obtained from the University of Sydney's Human Research Ethics Committee (Project number: 2022/493).

### Survey

The survey was divided into three sections that covered demographic information, KAP, and workplace culture. Demographic questions included gender, age range, degree, years of experience, and CT experience. There were eight workplace culture questions, which focused on access to IPC equipment and risk desensitisation.

The KAP questions were based on NHMRC guidelines related to hand hygiene, infection control with CT equipment, and infection control with contrast tubing (Hill et al. 2023. Infection, prevention, and control in computer tomography: methodological principles for a national survey in knowledge, attitudes, and practices. [Manuscript submitted for publication]). [16] There were 10 knowledge and practice questions, and 11 attitudes questions. Each knowledge question had a corresponding attitudes and practice question. The knowledge questions required a true or false response, and correct responses were scored 1 point and incorrect responses scored “0”. The range of scores was from 0 to 10. Attitude questions asked participants to choose an option relating to their level of agreement to a statement on a five-point Likert scale (‘strongly disagree’ to ‘strongly agree’), and scored from 1 to 5 points. A complete score of 5 was given to the most appropriate response. Hence, ‘strongly agree’ responses were allocated either a score of 5 or 1 depending on whether the statement was in the affirmative or negative. The range of scores was from 11 to 55 for all 11 questions. Practice question response options ranged from ‘never’ to ‘always’, which were also allocated 1 to 5 points. Two practice questions had more specific time frames (e.g. ‘once a day’, ‘between every patient’) and participants could choose from six options. The range of scores was from 10 to 52 taking into consideration both 5 and 6-likert scales.

### Data analysis

Demographic and descriptive data were presented as total number of individuals (n) with percentages of the total sample (%). Spearman’s correlation was used to compare the scores between knowledge, attitudes, and practice. The strength was determined by Cohen’s criteria, where higher scores indicate greater correlation between components [17]. The Kruskal–Wallis test was used to compare the KAP scores between demographic categories, and Mann–Whitney U was used as a post-hoc test. Pearson’s Chi Square was used to compare categorical data such as demographic data with workplace culture. The Statistical Package for the Social Sciences (SPSS) version 28 was used for all statistical analysis [18]. The p value was set to less than 0.05.

## Results

Of the 192 participants that started the survey, 147 completed all the survey components. Table 1 presents the demographic data of these participants. Of the 147 participants, 127 were radiographers (86%) and 20 were nurses (14%). Overall, the sample of radiographers that completed the survey were very experienced in CT, with 76% (97/127) having 6 or more years working in CT. Radiography and nurse participants were drawn from all states and territories of Australia, with 87% from the most populous states of New South Wales, Victoria and Queensland. Approximately 78% of the survey participants worked in a metropolitan area, 61% of whom worked at public hospitals and 39% from private workplaces.

**Table 1:** Demographic data of study participants (*n* = 147)

		Public (*n* = 89)	Private (*n* = 58)	Total (*n* = 147)
Profession	Radiographer	69	58	127 (86%)
	Nurse	20	0	20 (14%)
Gender	Female	67	40	107 (73%)
	Male	21	18	39 (27%)
	Prefer not to say	1	0	1 (1%)
Age (years)	20–25	8	6	14 (10%)
	26–30	19	8	27 (18%)
	31–35	15	12	27 (18%)
	36–40	14	9	23 (16%)
	41–50	21	7	28 (19%)
	50 +	12	16	28 (19%)
Years in Profession	< 1 year	1	2	3 (2%)
	1–5 years	20	8	28 (19%)
	6–10 years	18	12	30 (20%)
	10 + years	50	36	86 (59%)
Years in CT	< 1 year	10	2	12 (8%)
	1–5 years	24	14	38 (26%)
	6–10 years	21	11	32 (22%)
	10 + years	34	31	65 (44%)
State	Australian Capital Territory	6	1	7 (5%)
	New South Wales	31	29	60 (41%)
	Northern Territory	3	0	3 (2%)
	Queensland	3	13	16 (11%)
	South Australia	2	3	5 (3%)
	Tasmania	4	3	7 (5%)
	Victoria	39	8	47 (32%)
	Western Australia	1	1	2 (1%)
Region	Metropolitan	68	46	114 (78%)
	Rural	19	12	31 (21%)
	Remote	2	0	2 (1%)

### Knowledge, attitudes, and practice

**Fig. 1:**
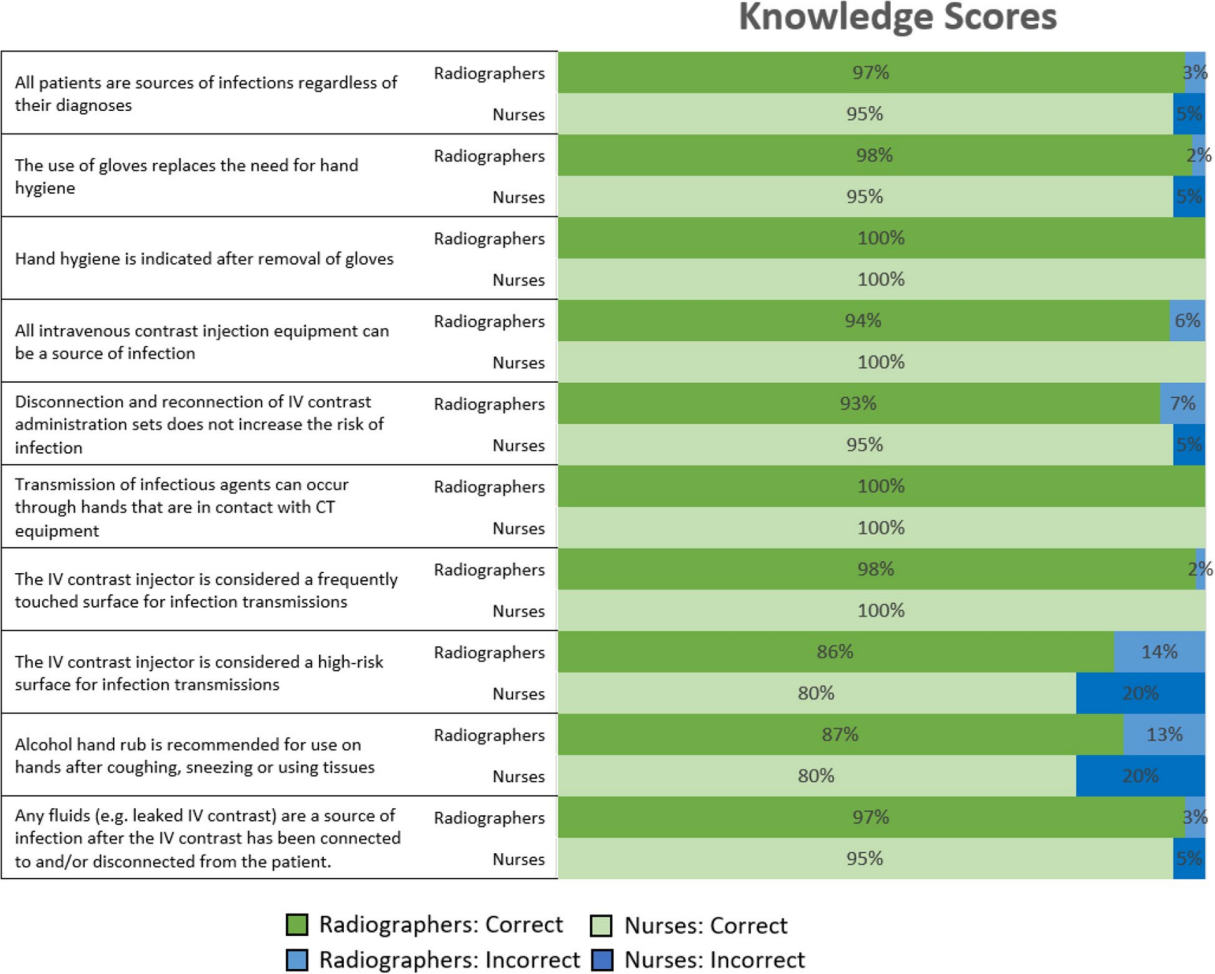
Knowledge questions and responses for radiographers (*n* = 127) and nurses (*n* = 20)

The main component of the survey was the KAP questions, and Fig. 1 presents the questions and the correct and incorrect responses of the radiographers and nurses for the knowledge component. For each knowledge question, agreement ranged between 80 to 100% for nurses, and 86 to 100% for radiographers. All participants agreed that contamination can occur from people touching CT equipment and that hand hygiene is required after removing gloves.

Table 2 presents the KAP total scores overall. The participants’ Likert scores were given values between 1–5, allowing percentages to be calculated. Since a score of 1 was given to the least favourable response, the range of possible scores for attitudes and practice were 11 to 55 and 10 to 52 respectively.

**Table 2:** Knowledge, attitudes, and practice scores of radiographers (*n* = 127) and Nurses (*n* = 20)

Section (Score Range)		Min	Max	Average ± Standard Deviation
Knowledge (0 to 10)	Radiographers	7.0 (70%)	10.0 (100%)	9.5 (95%) ± 0.7
	Nurses	8.0 (80%)	10.0 (100%)	9.4 (94%) ± 0.7
Attitudes (11 to 55)	Radiographers	36.0 (65%)	55.0 (100%)	47.8 (87%) ± 4.6
	Nurses	42.0 (76%)	55.0 (100%)	49.5 (90%) ± 4.7
Practice (10 to 52)	Radiographers	28.0 (54%)	52.0 (100%)	43.0 (83%) ± 5.1
	Nurses	39.0 (75%)	52.0 (100%)	46.0 (88%) ± 3.8

Both radiographers and nurses scored high in the knowledge section of the survey. There was no statistically significant difference between radiographers and nurses on their knowledge and attitudes. However, nurses had statistically higher practice scores than radiographers (*p* = 0.014). Spearman’s correlation was used to compare the relationship between the KAP scores, and Table 3 presents the results. Overall, there were positive correlations between KAP variables. There was a moderate positive correlation between knowledge and attitudes for both radiographers (rho = 0.394, *p* < 0.001) and nurses (rho = 0.410, *p* = 0.073) though the values were not statistically significant for nurses. Radiographers also had a moderate positive relationship between attitudes and practice (rho = 0.466, *p* < 0.001).

**Table 3:** Knowledge, attitudes, and practice correlation of radiographers (*n* = 127) and Nurses (*n* = 20)

		Spearman’s Rho	Strength	*p*-value
Knowledge vs Attitude	Radiographers	0.394	Moderate	< 0.001*
	Nurses	0.410	Moderate	0.073
Knowledge vs Practice	Radiographers	0.202	Weak	0.023*
	Nurses	0.256	Weak	0.276
Attitudes vs Practice	Radiographers	0.466	Moderate	< 0.001*
	Nurses	0.228	Weak	0.334

^*^p < 0.05

For the radiographer participants only (*n* = 127), KAP scores were compared with demographic qualities using the Kruskal Wallis and Mann–Whitney U test. There was no significance between KAP scores and radiographer’s age range, degree, position, years in profession, state, location, and years in CT.

CT radiographers who had an IPC team in their workplace (*n* = 74) had higher attitudes (*p* = 0.034) and practice (*p* = 0.012) scores than radiographers without an IPC team or who were unsure if they had an IPC team (*n* = 53). There was no statistical difference in their knowledge scores. All radiographers working in private institutions were compared with public hospital radiographers. Public hospital radiographers had statistically higher attitudes (*p* = 0.032) and practice (*p* = 0.044) scores than private practice and hospital staff but no difference in their knowledge scores.

### Workplace culture

**Fig. 2:**
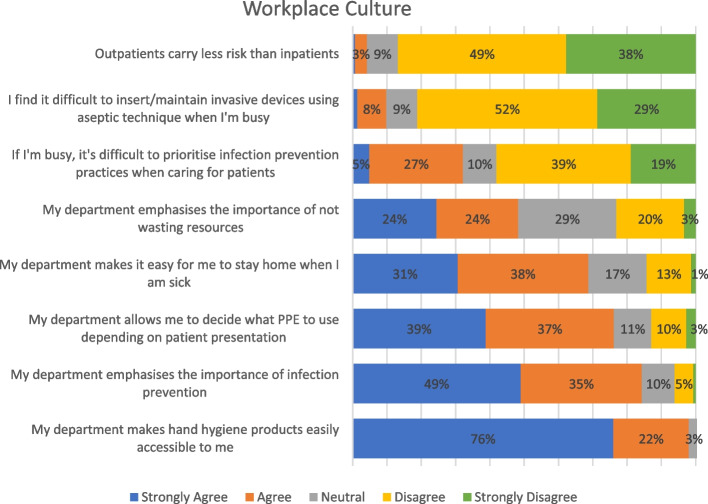
Workplace culture and infection prevention practices of radiographers and nurses *n* = 147

Participants were asked about their workplace, including their ability to adhere to IPC when they are busy (Fig. 2). Most participants agreed or strongly agreed that hand hygiene products were accessible, there was an emphasis on IPC in their workplace, and it was easy to stay home when they were sick. There were a moderate mix of agreement and disagreement regarding the emphasis on wasting resources and prioritising IPC when it’s busy.

Pearson’s Chi Square tests were used to compare radiographer workplaces (public or private) with workplace culture questions (*n* = 127) presented in Fig. 2. Participants were asked about the emphasis on not wasting IPC resources in their radiology department. There was a statistical difference between public and private radiographers, χ^2^(4) = 9.852, *p* = 0.043, and approximately 54% of radiographers from private institutions agreed/strongly agreed that their workplace emphasised not wasting resources, while only 41% from public hospitals agreed/strongly agreed. There was no difference between private and public radiographers on their perception of infection risk between inpatients and outpatient patients.

## Discussion

The study aimed to examine the KAP of CT radiographers and radiology nurses on standard precautions in the CT suite. Overall, radiographers and nurses had similar average scores for knowledge (95% and 94% respectively) and attitudes (87% and 90% respectively). Nurses were found to have statistically higher practice scores than radiographers. This could be due to IPC training differences between radiographers and nurses, but also due to the greater care-focused role that radiology nurses play while radiographers focus on technical related imaging tasks [19]. A study in Sweden assessed surface contamination in CT and found no or minimal bacterial cultures on cannulation trolleys which are commonly maintained by nurses, however, that same study found bacteria in the CT control rooms, which typically are not required to be as heavily cleaned as cannulation trolleys and may highlight poor hand hygiene practices by radiographers [20].

There was a moderate relationship between attitudes and practice for radiographers. Knowledge and attitudes scores had a positive correlation for both radiographers and nurses. Previous studies have reported greater regular adherence to IPC among healthcare workers when they saw value in IPC practices which came from increasing knowledge, training, and experience [12, 21]. Perceived risk to oneself, their family, or a sense of responsibility towards their patients also motivated staff to adhere to standard precautions [21].

Occupational burnout is another consideration for IPC, and a focus group conducted with nurses found that workload pressures would cause nurses to stray from their protocols [22]. Burnout among radiographers has been increasing and negatively impacts attitudes, productivity, and adherence to practice [8]. In the current study, a third of participants agreed that it is difficult to prioritise IPC practices when the CT department is busy. However, there were overwhelmingly positive responses regarding IPC emphasis in workplaces and access to personal protective equipment (PPE).

There were no significant differences between KAP scores and radiographer age range, degree, years in profession or CT, and position. This could be because the KAP survey focused on standard precautions, and staff are expected to always adhere to those precautions [1]. This finding is not unusual, and a study among Jordanian radiographers also reported that age and experience did not influence knowledge scores [10]. The results of this study concur with other published studies of nurses, including one with paediatric ICU nurses on their knowledge of IPC for catheter use, which reported no difference in knowledge between years of experience, years in speciality, position, or education [23]. Another study by Slater et al., among Australian nurses from five different hospital departments found that age, years of experience, and qualifications had no impact on IPC practice with needleless connectors [24].

Radiographers working in CT in workplaces with an IPC team had higher attitudes and practice scores than radiographers without an IPC team or were unsure if they had an IPC team. An Australian study among hospital nurses reported that both infection control staff and colleagues had positive impacts on their hand hygiene practice [25] and nurses reported that infection control consultants and senior nurses are most influential on their behaviour regarding correct cleaning of needleless connectors [24]. Our results are consistent with previous studies indicating that IPC teams do positively influence practice and have strong clinical application for interventions and staff leadership in the area of IPC in Australian CT departments.

Some literature suggest that larger institutions also have better adherence to standard precautions because they are more likely to have IPC teams [12]. Results from this study demonstrated that public hospital CT radiographers had higher attitudes and practice scores than CT radiographers working in private medical imaging centres. The survey used in this study was developed from national guidelines [9], hence there was an assumption that radiographers will have similar attitudes and practices regardless of their workplace. This difference could be the result of the influence and presence of the IPC teams that are incorporated in large health services and the nature of their work as they must apply standard and transmission-based precautions for both outpatients and inpatients. The workload and IPC measures are also different between public and private imaging centres, where facilities such as isolation rooms are not needed or applicable in private practices [13]. Our Australian study results aligned with those of a Jordanian study that reported radiographers working in the public sector had higher knowledge scores for IPC practice in radiology departments than radiographers in the private sector [10].

Whilst the risk of hospital acquired infections and exposure for staff differs between public and private imaging departments, national IPC policies including PPE apply to both inpatients and outpatients [1, 16]. In this current Australia-wide study, radiographers from private institutions reported a greater emphasis on not wasting PPE resources than radiographers in public hospitals, and results concur with the Dann and Sun study where radiographers in Western Australia reported that PPE use differs between private imaging centres and hospital settings [13]. This difference between public and private workplaces could also impact the higher attitudes and practice results of radiographers from public hospitals. Limited PPE negatively impacts adherence to standard precautions and easy access to hand hygiene products is important to facilitate regular hand washing practices [12, 21].

### Limitations

This study assessed the knowledge, attitudes, and practice of CT radiographers and nurses. There was a smaller number of nurses who completed the survey, none of whom were from private institutions which is in keeping with private practice caseload. This limits the generalisability of the nursing results. Most participants were from metropolitan areas, and though there were participants from every state, a greater distribution across the country would have improved the reliability of the results.

## Conclusion and implications for practice

The findings of this study found that radiographers and nurses had a good baseline knowledge of standard precautions using national guidelines and both knowledge and practice were positively correlated with attitudes for radiographers. Radiographers working in institutions with an IPC team or in public hospitals had better attitudes and practice scores than radiographers who did not. Nurses had higher practice scores than radiographers, while age, education, and years of experience did not impact on KAP scores. The results suggest that the presence of IPC teams promotes greater awareness of IPC and standard practice, and an important consideration for future improvements to radiographers’ attitudes and practices. Taken together, private practice radiographers are more at risk of IPC breaches due to PPE concerns and IPC culture and represents an opportunity for quality improvements in this area such as training, access to PPE, clear communication of policies and guidelines, and auditing. This study establishes a baseline of the KAP of radiographers and radiology nurses for IPC in CT. Further qualitative research is needed to explore the influences or justifications for adherence or breaches of IPC in CT.

## Data Availability

The datasets generated and/or analysed during the current study are not publicly available as per the conditions set by the University of Sydney’s Human Research Ethics Committee but are available from the corresponding author on reasonable request.
